# Exercise with a wearable hip-assist robot improved physical function and walking efficiency in older adults

**DOI:** 10.1038/s41598-023-32335-8

**Published:** 2023-05-04

**Authors:** Su-Hyun Lee, Jihye Kim, Bokman Lim, Hwang-Jae Lee, Yun-Hee Kim

**Affiliations:** 1 Department of Physical and Rehabilitation Medicine, Center for Prevention and Rehabilitation, Heart Vascular Stroke Institute, Samsung Medical Center, Sungkyunkwan University School of Medicine, Seoul, 06351 Republic of Korea; 2 WIRobotics, Yongin, 16942 Republic of Korea; 3 Robot Business Team, Samsung Electronics, Suwon, 16677 Republic of Korea; 4 Haeundae Sharing and Happiness Hospital, Pusan, 48101 Republic of Korea

**Keywords:** Health care, Engineering

## Abstract

Wearable assistive robotics has emerged as a promising technology to supplement or replace motor functions and to retrain people recovering from an injury or living with reduced mobility. We developed delayed output feedback control for a wearable hip-assistive robot, the EX1, to provide gait assistance. Our purpose in this study was to investigate the effects of long-term exercise with EX1 on gait, physical function, and cardiopulmonary metabolic energy efficiency in elderly people. This study used parallel experimental (exercise with EX1) and control groups (exercise without EX1). A total of 60 community-dwelling elderly persons participated in 18 exercise intervention sessions during 6 weeks, and all participants were assessed at 5 time points: before exercise, after 9 exercise sessions, after 18 sessions, and 1 month and 3 months after the last session. The spatiotemporal gait parameters, kinematics, kinetics, and muscle strength of the trunk and lower extremities improved more after exercise with EX1 than in that without EX1. Furthermore, the effort of muscles over the trunk and lower extremities throughout the total gait cycle (100%) significantly decreased after exercise with EX1. The net metabolic energy costs during walking significantly improved, and functional assessment scores improved more in the experimental group than in the control group. Our findings provide evidence supporting the application of EX1 in physical activity and gait exercise is effective to improve age-related declines in gait, physical function, and cardiopulmonary metabolic efficiency among older adults.

## Introduction

Life expectancy is increasing in most countries, and rapid population aging is being reported worldwide. Because the prevalence of gait disorders increases with age, the expected demographic changes will result in increased numbers of people affected in the coming decades. Elderly adults normally experience decreases in their physical capabilities due to deterioration of the neuromusculoskeletal system, which causes them to walk with a changed gait pattern and to become more cautious^1–3^. Increase in step variability and the metabolic cost of walking and decrease in preferred gait speed, step length, and range of motion of the ankle, knee, and hip joints have been reported as typical gait pattern changes associated with increased age^4,5^. In many cases, declines in physical conditioning and gait function with age ultimately lead to a sedentary lifestyle, which is strongly correlated with a variety of cardiovascular and metabolic diseases^6^. As life expectancy continues to increase worldwide, two major questions have arisen: (a) Will the increase in life span include several years of healthy life? and (b) How do we promote health-related quality of life into old age?^7^. The concepts of “aging well,” including “active-”, “successful-”, and “healthy aging”, have emerged in recent years to promote mental and physical health throughout aging^8,9^.

The risk of falls increases with age, and fall prevention among older adults is one of the most important public health issues in today’s aging society^10^. The risk of fall increases with age for many reasons. The most common causes of falls by elderly people are trips and slips, and the ability to rapidly move the entire leg is a potentially important aspect of preventing such falls^11^. Older adults generally redistribute joint moment and force across their lower extremities to maintain a gait performance similar to those of young people. Specifically, older adults tend to transfer the mechanical demands of walking on the lower extremity from distal to proximal, producing significantly more net positive work at the hip to compensate for less net positive work at the ankle^12^. The hip joint is vital for generating movement throughout the whole leg and is important for fall prevention^11^.

Regular physical activity and exercise are recommended as a primary countermeasure to the natural decline of physical function with age^13^. Participation in physical activity and exercise can help to maintain or improve health, physical function, and quality of life and reduce falls among elderly people in general and in those with morbidities in particular^14^. Improvements in mental health; psychological, emotional, and social well-being; and cognitive function are also associated with regular physical activity and exercise^8,15^. However, the reduced bodily functions of many older adults are a major barrier to regular execution of a standard exercise program^16–18^. Maintaining physical activity and exercise usually requires substantial support and supervision. Even then, many seniors fail to maintain such activity due to difficulties negotiating everyday participation, such as schedule conflicts and competing sedentary activities or health issues^19^. In addition, mobility limitations can be a challenge for many older adults trying to maintain an exercise program^20^. Therefore, alternative exercise programs providing regular physical activity to the elderly should be suggested.

Wearable assistive robotics has emerged as a promising technology to supplement or replace motor functions and to retrain people recovering from an injury or living with reduced mobility^21^. Robotic assistance has the potential to assist locomotion in a variety of areas, including recreation, clinical rehabilitation and assistance, and workplaces^22,23^. The use of wearable assistive devices has become one of the most promising ways to assist individuals with gait disorders^16,24,25^. Robot-assisted rehabilitation can reduce the heavy physical burden otherwise placed on therapists and can provide long training sessions with good consistency^26,27^. Several studies have reported that robot-assisted gait training is effective for improvement of gait and functional capability in people with mobility impairment^28–32^. In a study using hybrid Assistive Limb (HAL), HAL-assisted gait training significantly improved gait ability after intervention and the effect was maintained for 3 months after the training in chronic stroke patients^28^. Ogino et al. suggested that gait training using Gait Exercise Assist Robot is are effective for improving gait ability than treadmill gait training among subjects with chronic stroke^29^, and a study using Honda Walking Assistive device (HWA) reported that HWA-assisted gait training was safe and feasible, and could be effective for the elderly improvement of gait ability, hip function, and gait pattern after total hip arthroplasty^30^. Furthermore, a study using Alter G Bionic Leg Orthosis demonstrated that home-based robot-assisted gait training program could elicit improvements in clinical functional outcomes in patients with stroke and the effect was maintain for 22-week assessment^32^.

Robotic hip exoskeletons are human–robot cooperation systems that integrate robot power with human intelligence^33^. They can train the wearer’s muscles and assist movement by providing controllable assistive force/torque at the hip joint. They can also improve strength and endurance^1^. Compared with other human joints, the hip joint imposes higher metabolic costs to generate similar mechanical joint power due to differences in muscle characteristics^34^. Therefore, a robotic hip exoskeleton is a promising strategy for increasing the efficiency of human walking because the human hip joint produces large torque during the activities of daily living^35^.

**Figure 1:**
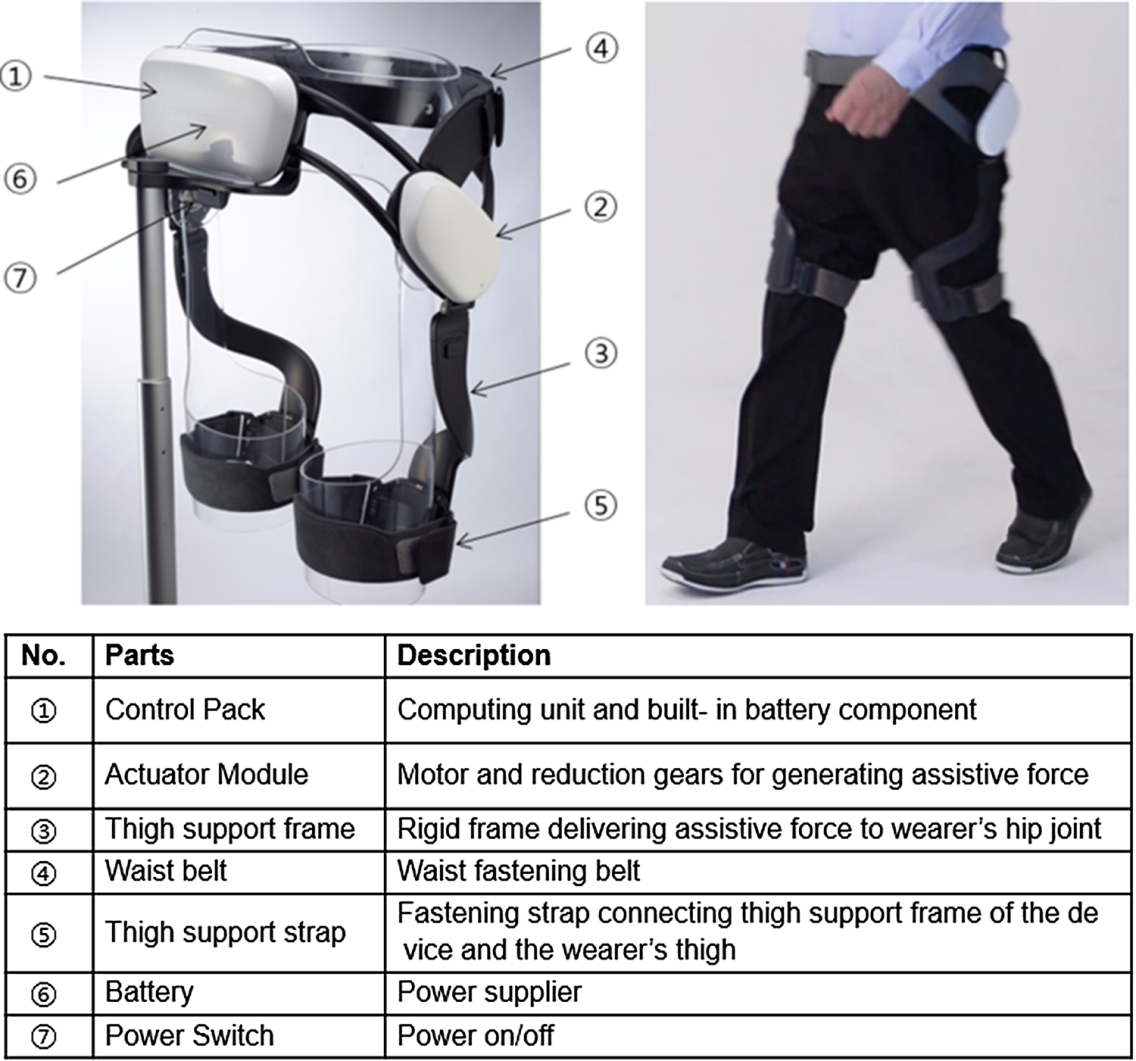
EX1 and description of the exterior.

We developed a robotic hip exoskeleton, the EX1 (Fig. 1), and a delayed output feedback control (DOFC) (see Supplementary Figs. S1–S3 online) to provide gait assistance. In our previous studies, we demonstrated that EX1 decreased the metabolic energy cost and improved gait efficiency in older adults during overground walking and stair climbing^25,36,37^. To further our investigation into the effectiveness of EX1, we conducted this study to demonstrate the long-term effect of EX1 use. Our goal was to determine whether task-specific physical activity and functional gait exercise with EX1 would be effective in improving spatiotemporal parameters, kinematics, kinetics, muscle effort, metabolic demand, and physical function in older adults compared to exercise without EX1.

## Results

### Effects of EX1 exercise on spatiotemporal gait parameters, kinematics, and kinetics

The specific values for spatiotemporal gait parameters, joint kinematics, and kinetics at the pre-exercise (Pre), post-exercise (Post), and 1-month and 3-month follow up (1 m FU and 3 m FU) time points after exercise are presented in Table 1 and Supplementary Figs. S8–S10 online. The experimental group showed a significant change in the spatiotemporal gait parameters (gait speed, cadence, and stride length) from Pre to all other time points (P < 0.01), but the control group showed significant improvement in the spatiotemporal gait parameters only from Pre to Post (P < 0.05). In addition, hip joint range of motion (ROM) improved significantly from Pre to all other time points (P < 0.01), and ankle joint ROM improved significantly from Pre to Post (P < 0.05) in the experimental group. Peak hip extension, ankle plantarflexion moment, and the second peak of vertical ground reaction force changed significantly from Pre to Post after exercise with EX1 (P < 0.05). Furthermore, significant group × time interactions were found in gait speed, cadence, and hip joint ROM, and the experimental group demonstrated greater improvement than the control group (P < 0.05).

**Table 1:** Effects of EX1 exercise on spatiotemporal gait parameters, kinematics, and kinetics.

	Experimental group				Control group				Between groups
	Pre	Post	1 m FU	3 m FU	Pre	Post	1 m FU	3 m FU	P value
Spatiotemporal gait parameters									
Gait speed (m/s)	1.18 (0.16)	1.35 (0.14)**	1.34 (0.13)**	1.32 (0.14)**	1.17 (0.12)	1.26 (0.13)*	1.23 (0.10)	1.22 (0.11)	0.005
Cadence (step/min)	118.26 (8.47)	125.54 (7.92)**	125.35 (9.73)**	124.92 (9.20)**	118.03 (8.25)	122.05 (6.18)*	120.29 (6.31)	119.53 (7.59)	0.036
Stride length (m)	1.20 (0.14)	1.29 (0.11)**	1.28 (0.10)**	1.27 (0.10)**	1.19 (0.11)	1.24 (0.12)*	1.23 (0.09)	1.22 (0.11)	0.125
Joint range of motion (degree)									
Hip	41.35 (4.62)	46.69 (7.51)**	45.12 (3.51)**	44.42 (4.71)**	39.67 (5.69)	40.73 (8.82)	40.82 (5.04)	41.60 (4.08)	0.001
Knee	60.94 (5.73)	62.07 (4.23)	61.08 (4.86)	61.74 (5.06)	59.71 (5.96)	61.24 (6.56)	60.73 (4.88)	61.14 (5.46)	0.521
Ankle	26.15 (3.21)	29.46 (5.94)*	28.10 (4.06)	27.29 (4.59)	25.47 (4.01)	26.68 (5.51)	26.97 (3.70)	26.80 (3.38)	0.139
Joint peak moment (N m/kg)									
Hip flexion	− 0.61 (0.24)	− 0.76 (0.34)	− 0.76 (0.23)	− 0.71 (0.36)	− 0.65 (0.24)	− 0.78 (0.32)	− 0.74 (0.25)	− 0.71 (0.28)	0.814
Hip extension	0.85 (0.36)	1.09 (0.30)*	0.86 (0.31)	0.99 (0.44)	0.93 (0.45)	0.91 (0.42)	0.95 (0.35)	1.00 (0.34)	0.982
Knee flexion	− 0.21 (0.10)	− 0.22 (0.09)	− 0.23 (0.07)	− 0.25 (0.15)	− 0.24 (0.16)	− 0.25 (0.12)	− 0.26 (0.11)	− 0.26 (0.09)	0.162
Knee extension	0.43 (0.22)	0.58 (0.22)	0.54 (0.27)	0.58 (0.31)	0.51 (0.24)	0.54 (0.29)	0.48 (0.23)	0.58 (0.31)	0.407
Ankle PF	1.04 (0.21)	1.19 (0.20)*	1.12 (0.21)	1.11 (0.22)	1.15 (0.28)	1.14 (0.23)	1.15 (0.23)	1.11 (0.22)	0.523
Vertical ground reaction force (% body weight)									
1st peak	10.21 (4.42)	12.55 (4.47)	10.36 (4.22)	10.78 (4.80)	11.03 (6.31)	11.57 (7.92)	11.35 (5.24)	10.36 (6.60)	0.920
2nd peak	10.12 (4.47)	12.99 (4.52)*	11.56 (4.21)	10.26 (4.41)	11.13 (6.57)	10.53 (4.58)	10.33 (4.40)	9.87 (7.18)	0.432

Data are expressed as mean (standard deviation).

*FU* follow up, *PF* plantarflexion.

*Significant change compared with Pre (P < 0.05), **Significant change compared with Pre (P < 0.01).

### Effects of EX1 exercise on muscle strength (MVC) and muscle effort (%MVC)

**Figure 2:**
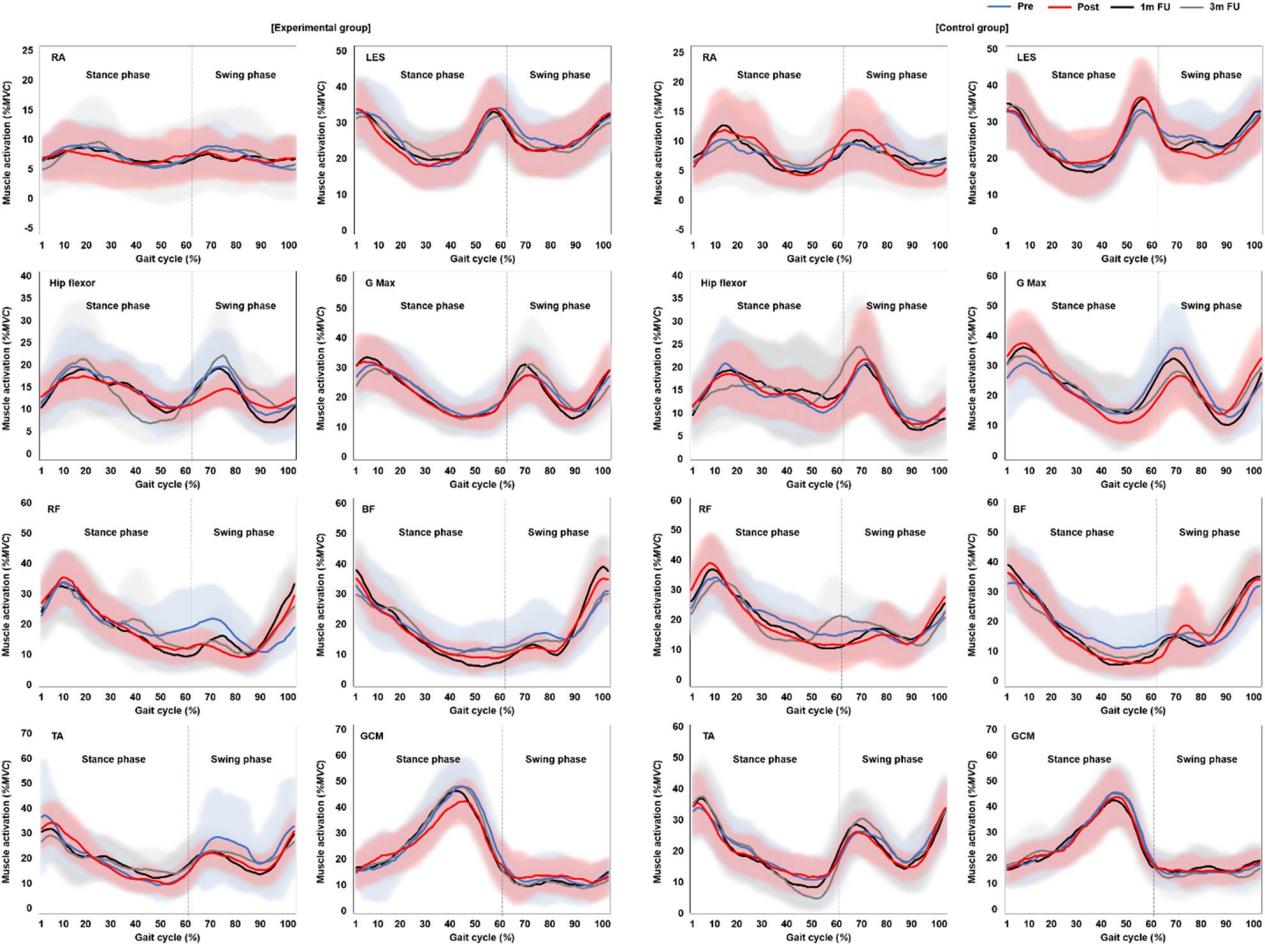
Muscle effort. Diagrams showing differences in the mean normalized muscle effort (%MVC) measured by surface electromyography during the gait cycle (%) at the Pre, Post, 1 m FU, and 3 m FU time points in the experimental and control groups. Muscle effort during the total gait cycle (100%) decreased significantly in the RA, LES, hip flexor, RF, BF, TA, and GCM after EX1 exercise (P < 0.05, P < 0.01), and those decreases were maintained to the 1 m and 3 m FU tests. The control group showed significant muscle effort decreases only in the RF and TA from Pre to Post (P < 0.05, P < 0.01). See Supplementary Table S1 for numerical data. *FU* follow up, *MVC* maximum voluntary contraction, *RA* rectus abdominis, *LES* lumbar extensor spinae, *G Max* gluteus maximus, *RF* rectus femoris, *BF* biceps femoris, *TA* tibialis anterior, *GCM* gastrocnemius medialis.

Figure 2, Supplementary Fig. S11 online, Supplementary TableS1, and Supplementary Table S2 online present the specific values for maximum voluntary contraction (MVC, μV) and muscle effort (%MVC) at the respective Pre, Post, 1 m FU, and 3 m FU time points. The experimental group showed significant improvements in the MVC of the rectus abdominis (RA), lumbar extensor spinae (LES), hip flexor, rectus femoris (RF), tibialis anterior (TA), and gastrocnemius medialis (GCM) after EX1 exercise, and the MVC changes of RA, hip flexor, RF, TA, and GCM remained significant up to 1 m FU (P < 0.05). However, the control group showed significant changes only in the MVC of the RA and RF from Pre to Post (P < 0.05). In addition, significant group × time interactions were found in the MVC of the RA, LES, hip flexor, gluteus maximus (G Max), TA, and GCM, and the experimental group demonstrated greater improvement than the control group. Muscle effort during the total gait cycle (100%) decreased significantly in the RA, LES, hip flexor, RF, biceps femoris (BF), TA, and GCM after EX1 exercise (P < 0.05, P < 0.01). The decreases in RA, RF, and BF were maintained to the post test, decreases in hip flexor and TA were maintained to the 1 m test, and decreases in LES and GCM were maintained to the 3 m test. In contrast, the control group showed significant muscle effort decreases only in the RF and TA (P < 0.05, P < 0.01). Significant group × time interactions were also found in muscle effort in the RA, and the experimental group demonstrated greater change than the control group (P < 0.05).

### Effect of EX1 exercise on metabolic demand

**Figure 3:**
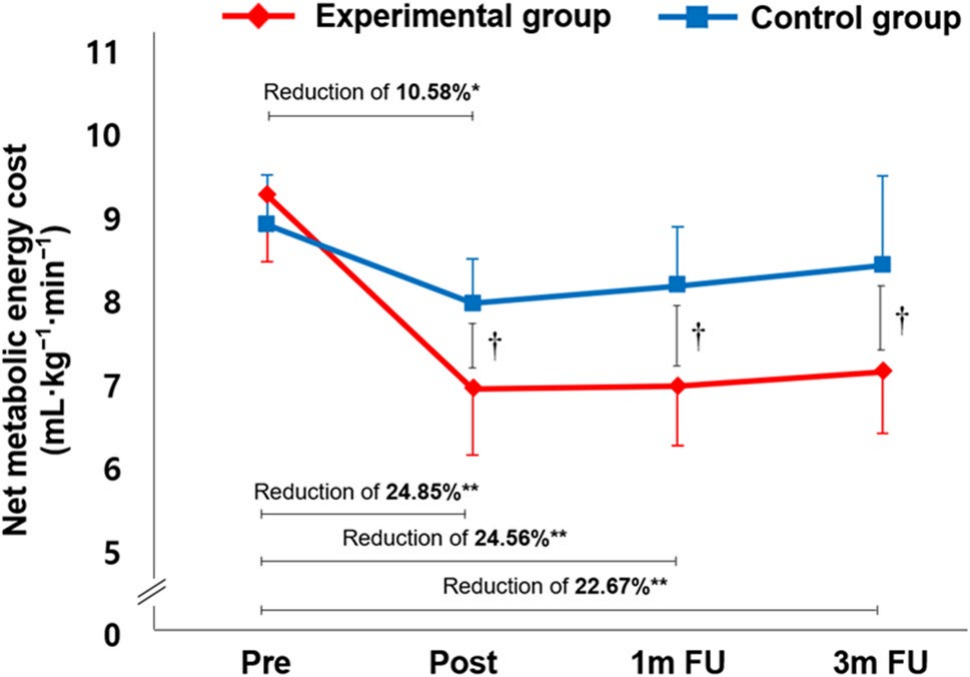
Metabolic energy costs. Changes in the net metabolic energy cost (mL/kg///min) at the Pre, Post, 1 m FU, and 3 m FU time points in the experimental and control groups. *FU* follow up. ^*^Significant change compared with Pre (P < 0.05), **Significant change compared with Pre (P < 0.01). ^†^Independent t test between two groups at each time point (P < 0.05).

The changed values for net metabolic energy cost (mL/kg/min) are shown in Fig. 3. The experimental group showed significant change in net metabolic energy cost from Pre to all other time points (P < 0.01): by 24.85% at Post, 24.56% at 1 m FU, and 22.67% at 3 m FU. The net metabolic energy cost of the control group also changed significantly from Pre to Post (P < 0.05): by 10.58% at Post, 8.32% at 1 m FU, and 5.53% at 3 m FU. The experimental group showed greater improvement than the control group at Post, 1 m FU, and 3 m FU (P < 0.05). Net metabolic energy cost also demonstrated a significant group × time interaction, indicating that gait exercise with EX1 was better than without EX1 for improving cardiopulmonary metabolic efficiency (P < 0.05).

### Functional assessment

**Figure 4:**
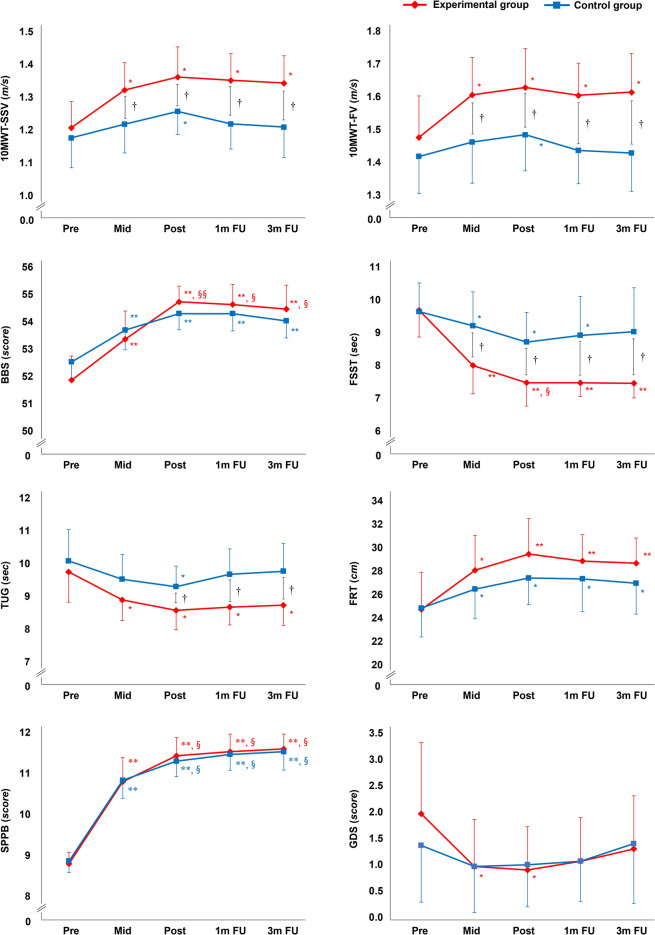
Functional outcomes. Changes in functional outcome at the Pre, Mid, Post, 1 m FU, and 3 m FU time points within the experimental and control groups. *FU* follow up, *10MWT-SSV* 10-Meter Walk Test for self-selected walking velocity, *10MWT-FV* 10-Meter Walk Test for fastest walking velocity, *BBS* Berg Balance Scale, *FSST* Four Square Step Test, *TUG* Timed Up and Go, *FRT* Functional Reach Test, *SPPB* Short Physical Performance Battery, *GDS-SF* Geriatric Depression Scale-Short form. *Significant change compared with Pre (P < 0.05), **Significant change compared with Pre (P < 0.01). ^§^Significant change compared with Mid (P < 0.05), ^§§^Significant change compared with Mid (P < 0.01). ^†^Independent t test between two groups at each time point (P < 0.05).

Figure 4 illustrates the progression of functional outcome scores at Pre, after 9 exercise sessions (Mid), Post, 1 m FU, and 3 m FU in the experimental and control groups. The experimental and control groups did not differ at Pre in any raw outcome measure, confirming that baseline conditions were similar between groups. Time had a main effect on all outcome measures except the Geriatric Depression Scale-Short form (GDS-SF) in the control group. Post hoc analyses revealed that the scores of the experimental group in the 10-Meter Walk Test for self-selected (10MWT-SSV) and -fastest walking velocities (-FV), Berg Balance Scale (BBS), Four Square Step Test (FSST), Timed Up and Go (TUG), Functional Reach Test (FRT), and Short Physical Performance Battery (SPPB) changed from Pre to all other time points (P < 0.05, P < 0.01). The control group changed from Pre to all other time points in the BBS, FRT, and SPPB (P < 0.05, P < 0.01). The experimental group also changed from Mid to Post, 1 m FU, and 3 m FU in the BBS and from Mid to Post in the FSST (P < 0.05, P < 0.01). Both groups changed from Mid to Post, 1 m FU, and 3 m FU in the SPPB (P < 0.05).

Repeated measures ANOVA revealed significant group × time interactions in the 10MWT-SSV and -FV, FSST, and TUG (P < 0.05). The experimental group showed greater improvement than the control group in the 10MWT-SSV and -FV and FSST at Mid, Post, 1 m FU, and 3 m FU (P < 0.05) and in the TUG at Post, 1 m FU, and 3 m FU (P < 0.05).

## Discussion

Our findings in this study suggest that task-specific physical activity and functional gait exercise with EX1 have several key advantages for gait, physical function, and metabolic energy efficiency over exercise without EX1. Spatiotemporal gait parameters, kinematics, and kinetics improved significantly after EX1 exercise in the experimental group, and changes in spatiotemporal gait parameters (gait speed, cadence, and stride length) and hip joint ROM remained significant up to 3 m FU. In addition, the experimental group showed significant improvements in the MVC of the RA, LES, hip flexor, RF, TA, and GCM after EX1 exercise, and the MVC changes of RA, hip flexor, RF, TA, and GCM remained significant up to 1 m FU. The decreases of muscle effort in RA, RF, and BF were maintained to the post test, decreases of muscle effort in hip flexor and TA were maintained to the 1 m test, and decreases of muscle effort in LES and GCM were maintained to the 3 m FU. Furthermore, the experimental group showed significant change in net metabolic energy cost and the scores in 10MWT-SSV, 10MWT-FV, BBS, FSST, TUG, FRT, and SPPB from Pre to all other time points.

Within the natural processes of aging, decreases in gait speed, stride length, cadence, and relative duration of the swing phase as well as increases in gait speed variability and single- and double-support time are common and expected^38–40^. Mobility and physical function impairments, often operationalized as spatiotemporal gait parameters, are strongly associated with concerns about falling. A decrease in gait speed is frequently used as a predictor of fall risk and disability and a marker of frailty because it correlates with functional loss and imminent death^41,42^. Therefore, spatiotemporal gait parameters are a major factor influencing mobility and physical function, and they are meaningful biomechanical assessments for monitoring the effects of exercise programs in older adults. According to our results, gait speed, cadence, and stride length improved significantly after EX1 exercise in the experimental group, and those improvements were maintained at the 1 m and 3 m FU time points after cessation of exercise. In contrast, the control group showed significant improvements in the spatiotemporal parameters after exercise, but those improvements were not maintained at the 1 m or 3 m FU assessment. These results lead us to postulate that task-specific physical activity and functional gait exercise with EX1 can improve spatiotemporal gait parameters and offer long-term health benefits. Several studies have shown that changes in certain spatiotemporal gait parameters such as velocity or stride length increase fall risk^43–45^, and aging is associated with an increased risk of falls^46^. Improvements in spatiotemporal parameters maintained in the FU assessments after EX1 exercise support that EX1 exercise has the ability to reduce the risk of fall. Furthermore, by providing appropriate levels of assistance, EX1 can provide extensive, repetitive, task-specific gait exercise that can facilitate durable improvement in spatiotemporal parameters. Our results demonstrate the superiority of repetitive EX1 exercise over that without EX1 in terms of the durability of improvements in spatiotemporal gait parameters.

Age-related changes in biomechanical gait characteristics are associated with limited lower-limb joint ROM and significant reductions in joint moment and force as a result of physiological and neuromuscular changes^47–49^. It is especially important to identify changes in joint movement angles when assessing the gait performance of elderly people. Biomechanical analyses of lower extremity activity in elderly people have shown decreased ROM of the hip, knee, and ankle joint in the sagittal plane during walking tasks^50–52^. That decrease is associated with changes observed in kinetic data for those joints. In particular, elderly people showed reduced hip extension moment at the loading phase of gait^53^ and plantar flexion peak moment of the ankle joint in the late stance phase compared with young persons^54^. Elderly individuals also demonstrate limited capacity to advance the limb during the push-off period due to decreased plantar flexor moments and force^55^. In our study, hip joint and ankle joint ROM significantly improved in the experimental group after EX1 exercise, and changes in hip joint ROM remained significant up to 3 m FU. The peak hip extension and ankle plantarflexion moment and the second peak of vertical ground reaction force also changed significantly from Pre to Post in the experimental group. In contrast, the control group showed no significant change in joint ROM, peak moment, or vertical ground reaction force. The occurrence of falls in older adults is related to various factors, such as balance disorders, unstable lower limbs, and biomechanical and neuromuscular changes^56^. These results indicated that EX1 exercise could increase lower-limb joint ROM, joint moment, and force. In other words, EX1 exercise can be recommended to help prevent falls by reducing age-related changes in biomechanical gait characteristics.

Older adults generally have plantar flexor muscle weakness, which could cause a weak push-off power^57^. During the late stance phase, decreased push-off power could affect trunk stabilization and trunk progression, which cause a shorter step length in elderly people^58^. As a potential compensation for reduction of ankle push-off power, older adults rely more on the hip musculature for power generation than young adults, a phenomenon known as *distal-to-proximal redistribution*^59^. This redistribution could explain, at least in part, why elderly people pay greater metabolic energy costs for walking than younger people and might be considered maladaptive^60^. The functional consequences of higher metabolic energy costs include reduced physical activity and independence, gait inefficiency, and lower quality of life^61^. In our study, the experimental group showed significant improvements in muscle strength of the trunk (RA, LES) and lower extremities (hip flexor, RF, TA, and GCM) after EX1 exercise, and the changes in muscle strength of RA, hip flexor, RF, TA, and GCM remained significant up to 1 m FU. Thus, EX1, which provides assistance torque only to the hip joint, affects not only the strength of hip joint muscles, but also the strength of trunk and ankle muscles. Older adults have been reported to rely less on ankle strategies for balance, resulting in greater use of hip strategies^62^. We assume that EX1 exercise is effective in improving both ankle and hip strategies, along with strength of the muscles around these joints in older adults. Furthermore, the experimental group showed significant changes in the muscle effort of the trunk (RA, LES) and lower extremities (hip flexor, RF, BF, TA, and GCM) during the total gait cycle (100%) after EX1 exercise. Decreases of muscle effort in hip flexor and TA were maintained to the 1 m test, and decreases of muscle effort in LES and GCM were maintained to the 3 m FU. The reduced muscle effort during walking demonstrated that EX1 exercise is effective. The experimental group demonstrated a greater than 20% net metabolic decrease when walking. As such, decreased walking energy cost is predicted to significantly improve health, quality of life, and life expectancy in older adults^6^. Our results indicated that EX1 exercise could increase muscle strength and help older adults to walk more efficiently by reducing the muscle effort required by their gait, which could lead to a reduction in cardiopulmonary metabolic costs. In other words, the gait inefficiency common in older adults could be improved through EX1 exercise.

Gait and balance disorders are common causes of falls by elderly people, and falls often lead to injuries, disability, loss of independence, and low quality of life^63^. Both dynamic and static balance are key components in many activities of daily living, from simple activities such as quiet standing to more complex activities such as walking while talking or changing direction. As people age, their ability to control their balance and gait function deteriorates due to alterations in the visual, vestibular, somatosensory, musculoskeletal, and central nervous systems^64,65^. Therefore, specific exercise programs to improve age-related changes in balance and gait function should be suggested for older adults. In this study, we conducted functional assessments of gait and balance to examine how EX1 exercise affected elderly people. In addition, participants completed the GDS-SF to monitor changes in depression after exercise. We found greater improvements in all the functional assessments after exercise with EX1 than without EX1. Previous studies have shown that better physical function is generally related to a lower incidence of depressive symptoms among older adults^66,67^. It is also associated with better mental health, well-being, and quality of life^68^. In this study, only the experimental group, which showed great improvement in functional assessments after exercise with EX1, showed a significant change in the GDS-SF. Improved physical functioning after EX1 exercise might have a positive effect on depression in elderly people.

Globally, several robot-assisted gait devices have been developed, and the research into rehabilitation robotics has increased. Pre-clinical studies with robotic hip exoskeletons have demonstrated improvements in gait and physical function, muscle effort, and cardiopulmonary metabolic efficiency in elderly individuals and individuals with stroke^16,24–27,31,32,60^. A study using an exoskeletal Active Pelvis Orthosis (APO)^16^ investigated its feasibility for cardiopulmonary gait training in the elderly and showed a decrease in Metabolic Cost of Transport (− 26.6 ± 16.1%) during treadmill walking after a 4-week APO-assisted training program, similar to our results. Our prior study with stroke subjects using an earlier version of the EX1^24^ demonstrated improvement in spatiotemporal gait parameters and muscle efforts and decrease in net cardiopulmonary metabolic energy cost (− 14.71%) after the training intervention. Furthermore, a study using a soft robotics suit^69^ reported the long-term rehabilitation effectiveness of the robotic suit in elderly persons and showed a improved gait characteristics and an increased walk ratio with an average of 9.8% compared with the initial state. While the exercise program of these studies consisted only of treadmill and overground walking, the intervention session in the present study consisted of long-term task-specific physical activity and functional gait exercise that elderly people can perform in their daily lives, including obstacle avoidance and navigation, multidirectional stepping, walking on varied surfaces, stair climbing, and community mobility. Therefore, our findings support the adoption of EX1 not only for training and rehabilitation to facilitate active aging in the elderly, but also for physical activity and gait exercises in daily life for community dwelling elderly. In addition, the maintained effect of EX1 exercise could be suggested the EX1-assisted exercise can promote sustained improvement in gait and functional abilities and can be recommended as a community exercise program.

This study has limitations related to inadequate control of everyday activity level of the participants beyond the experimental session. During the long-term intervention and follow-up period, other factors that might have affected the study outcome, such as an individual’s overall amount of physical activity, were not controlled or monitored. All participants were allowed to perform their usual daily activities and completed the study without drop-outs or intervention- or fall-related injuries during the study period. We believe that this limitation should not significantly affect our results. An additional limitation is related to sample size. The statistical power of this study was low because of the small number of participants; therefore, our results cannot be generalized to the entire elderly population. Nevertheless, this study demonstrated that use of EX1 in addition to task-specific physical activity and functional gait exercise produced better gait, physical function, and gait efficiency outcomes than similar exercise without EX1.

This study is the first randomized controlled trial to consider gait, physical function, and gait efficiency in elderly people after long-term, task-specific, and functional gait exercise with a wearable robot. Our findings provide evidence supporting the application of EX1 to regular physical activity and exercise to improve age-related declines in gait and physical functioning. All participants in this study completed protocols with EX1 without any specific adverse events, indicating that EX1 exercise is safe and does not pose risks to apply for older adults. Nonetheless, several participants suggested improvements to the weight, noise, and design of EX1. Future works will examine the usability of and satisfaction with EX1.

## Methods

### Participants

A total of 60 subjects was included in this study, and their characteristics are shown in Table 2. Based on history taking and functional assessment, we excluded individuals with a history of neurological disorders and musculoskeletal disorders that affect walking capacity, efficiency, and endurance. The eligible participants were randomly assigned to either the experimental (exercise with EX1, n = 30) or control (exercise without EX1, n = 30) group using a computer-generated 1:1 allocation. The study procedures were approved by the ethics committee of the Samsung Medical Center Institutional Review Board (Approval Number: 2019-04-063) and registered with ClinicalTrial.gov (NCT03962517). Written informed consent was obtained from all participants before they entered the study, and all methods were carried out in accordance with the approved study protocol.

**Table 2:** Baseline characteristics of participants.

Characteristic	Experimental group	Control group	*P* value
Sex (male/female)	13/17	14/16	0.795
Age, years	75.93 (5.53)	75.73 (4.10)	0.874
Height, cm	158.97 (6.33)	160.43 (8.22)	0.442
Weight, kg	60.93 (8.09)	61.17 (9.49)	0.919
Body mass index, kg/m^2^	24.09 (2.65)	23.72 (2.74)	0.592
Self-selected treadmill speed, km/h	1.97 (0.46)	1.98 (0.45)	0.977

Data are expressed as mean (standard deviation).

### Experimental procedure and study design

#### Overview

This study used a repeated-measure, single-blinded (evaluator), randomized, controlled, and parallel design. All participants attended 23 experimental visits: 5 testing visits (Pre, Mid, Post, 1 m FU, and 3 m FU) and 18 exercise visits.

#### Exercise

All participants performed task-specific physical activity and functional gait exercise, including obstacle avoidance and navigation, multidirectional stepping, walking on varied surfaces, stair climbing, and community mobility (walking in the hospital community) with EX1 (experimental group) or without EX1 (control group)^70^. The exercise intervention was conducted at a perceived exertion between 12 and 16 on Borg’s Rating of Perceived Exertion (RPE) scale (somewhat hard ~ hard)^71^ for 6 weeks, with 3 exercise sessions per week (18 sessions total). The duration of each exercise session was 60 min: 5 min of warm-up, 50 min of exercise with 5 min of rest, and 5 min of cool-down. Exercise intensity was assessed with verbal questions twice during the intervention session based on the Borg RPE scale and was controlled by giving the subject a rest period and adjusting assistive torque of EX1 applied to the subject. For subject safety, a physical therapist supervised the intervention. If a participant missed an exercise session, an additional session was offered at another time of the week or at the end of the intervention period.

#### Testing

Participants were assessed at 5 time points: Pre, Mid, Post, 1 m FU, and 3 m FU after the last exercise session. Each assessment evaluated spatiotemporal gait parameters, kinematics, kinetics, muscle activity, cardiopulmonary metabolic energy efficiency, and functional outcomes (performance-based and subject-reported measures) except the Mid time point, at which only the functional outcomes were measured. The participants were asked to walk at a comfortable, self-selected speed along a 10-m walkway, and spatiotemporal, kinematic, kinetic, and muscle activity data were collected simultaneously. Cardiopulmonary metabolic energy efficiency was measured before placement of the sensors for participant comfort during 6 min of treadmill walking. All outcome measures were collected when subjects were not wearing the EX1 device.

Spatiotemporal and kinematic data were measured using a 3D motion capture system with 8 infrared cameras (Motion Analysis Corporation, Santa Rosa, CA, USA), and kinetic data were obtained using 2 force plates (TF-4060-B, Tec Gihan, Kyoto, Japan) embedded midway along the walkway. We collected the trajectories of 19 markers placed on anatomical landmarks using the Helen Hayes marker model^72^. The motion capture system allowed us to define each marker during collection, so marker position was recorded in real time. A standing calibration was used to obtain a rotation matrix for each limb segment and align the local (anatomical) reference frame for the thigh, shank, and foot to the global (laboratory) reference frame. Movement data were automatically converted to 3-dimensional coordinates with CORTEX version 6.2 motion capture software (Motion Analysis Corporation, Santa Rosa, CA, USA). Spatiotemporal, kinematic, and kinetic gait parameters were calculated for each gait cycle using Ortho Track 6.6.4 software (Motion Analysis Corporation).

To obtain surface electromyography (sEMG) signals (Noraxon USA Inc., Scottsdale, AZ, USA), bipolar surface electrodes (Ag/AgCl) were positioned on the right RA, LES, G Max, RF, BF, TA, and GCM, and the electrodes for the hip flexor were placed in the femoral triangle inferior to the inguinal ligament just medial to the rectus femoris. sEMG sensor placement was performed for all four assessments by the same professional therapist, following the recommendations of the Non-Invasive Assessment of Muscles Project (SENIAM)^73^ for reliable sensor placement. The skin was prepared for sEMG collection by shaving, abrading, and cleansing with alcohol to reduce surface impedance. In addition, footswitches (Model 500 DTS FootSwitch; Noraxon USA Inc., Scottsdale, AZ, USA) were placed on the right toe and heel to identify the timing of the stance and swing gait phases while walking.

Cardiopulmonary metabolic energy efficiency was measured using a validated and reliable portable telemetric gas analyzer system (K5, COSMED, Rome, Italy). The COSMED K5 portable cardiopulmonary metabolic system uses combined breath-by-breath technology to measure oxygen consumption (VO_2_) and carbon dioxide production (VCO_2_). The participants wore the system on the upper body with a face mask that prevented exposure to outside air for the breath analysis.

For the 10MWT-SSV and -FV measurements, subjects were asked to walk a total of 15 m at a comfortable and maximum speed, respectively^74^. The time it took to walk 10 m was measured, excluding the initial 2.5 m acceleration and the final 2.5 m deceleration, and the result in seconds was converted to speed (m/s). SPPB consists of three performance subtasks (balance, five repeated sit-to-stand on a chair cycles, and usual-pace walking speed) that measure balance, lower limb muscle strength, and mobility. The score of each of the three tests ranges from 0 to 4, where 4 indicates the best result and 0 the worst result, and a summary score ranges from a minimum of 0 points (worst performers) to a maximum of 12 points (best performers)^75^. BBS is a valid, qualitative, and efficient measure of balance measured through performance of functional activities that incorporate most components of postural control in older individuals. The tool consists of 14 items scored on an ordinal scale of 0 to 4, with 0 being the worst and 4 being the best performance of independent tasks for a total of 56 points^76^. For TUG measurement, subjects sit on a chair placed against the wall. They are instructed to stand up from a chair, walk at their normal pace on a 3 m pathway, turn around, walk back, and sit down on the chair again. The stopwatch is started on the word “go” and stopped when the subject sits down again^77^. FRT assesses subject stability by measuring the maximum distance (cm) an individual can reach forward while standing in a fixed base of support^78^, and GDS-SF consisting of 15 questions is a useful screening tool for monitoring mood status and evaluating depression^79^. All assessments were conducted by an experienced physical therapist blinded to intervention assignments.

### Wearable hip-assist robot, EX1

#### Hardware implementation

EX1, which was developed at Samsung Electronics Co., Ltd. (Suwon, Republic of Korea), is a lightweight, robotic exoskeleton designed to assist in the ambulatory function of elderly people. EX1 is designed based on a slim and backdrivable actuator^80^, and the latest version of the EX1 has been further reduced in weight by improving the structure of the wearable frame^81^. The device is worn around the user’s waist and thighs and assists with hip joint flexion and extension. The EX1 consists of snap-together components weighing 4.7 lb (2.1 kg) in total. The waist part houses 2 actuator modules, a Bluetooth module, and a control pack. Each actuator module houses a motor, an embedded angular position sensor, and a controller. The control pack contains a central processor, an inertial measurement unit sensor, a rechargeable battery pack, and a power switch. The torque assistance is transmitted to the user’s thighs via thigh frames. The trained physical therapist who operates the device can change the assist settings through software that runs on a mobile device.

#### Performance specification

EX1 defines the angle of the hip joint to be 0° when the user is wearing the device and standing upright. The hip flexion angle is increased up to 70° during stair climbing^82^. While the user is sitting on a chair, the hips need to be bent up to 100°. Therefore, the flexion angle of the device is mechanically limited to 110°, and the extension angle is set to 45°. However, a flexion angle of 100° and extension angle of 40° are set with the software to ensure the safety of both the users and the device. The device is torqued off when the user’s leg angle exceeds the range. Furthermore, the device alerts the physical therapist by sending a warning message to the mobile application when the joint angle reaches the limit.

The maximum assistance torque of the device is about 12 Nm. Furthermore, maintaining torque tracking performance is essential to deliver sustained hip power to the wearer during walking. The hip angular velocity measured by the device is 4–5 rad/s when the user is walking at 3–5 km/h. The device can provide torque assistance up to a maximum hip speed of 6 rad/s to accommodate faster swing speeds. For example, when the hip swing velocity needs to be increased to avoid obstacles, the torque tracking performance is ensured up to 6 rad/s (Supplementary Fig. S7). Since the EX1 can walk for 2 h continuously at 3 km/h, it can perform walking training for more than 1 h.

#### EX1 assistance algorithm and delayed output feedback control

We developed the DOFC, a time-delayed, self-feedback controller for gait assistance with EX1. By assigning the appropriate time-delay and self-feedback gain, we can generate stable assistive torque through interactions between the wearer and the exoskeleton. This control algorithm is simple but powerful because of its generalizability to changes in the user’s walking patterns. The assistance method changes the assistive torque according to gait pattern changes without requiring changes to the control parameters. Its implementation was first illustrated in^81,83^.

Most of the existing studies have focused on minimizing gait energy and strength loads and have been conducted with young and healthy subjects^84–87^. They have also focused on immediate effects rather than training effects. There are no tests that take into account gait training for more than 30 min in large numbers of older adults, as was done in this study. In elderly subjects with low muscle strength, torque greater than 10 Nm can cause gait instability, and some complained of discomfort due to the strong assistive force. Further research is needed to easily find the optimal torque level for elderly subjects with low muscle strength, and this is one of our future projects.

### Data analysis

Motion capture data were filtered with a Butterworth filter algorithm with a cutoff frequency of 6 Hz, 2-pass, fourth-order, and zero-phase shift filter. Hip, knee, and ankle moments were first calculated using inverse dynamics, kinematics, ground reaction forces applied to the foot, and the distance between the application of force and the center of mass of the segment. Moment data were scaled by body weight, and values for peak ankle plantarflexion moments and peak flexion and extension moments at the knee and hip were averaged across trials for each subject. The calculation of joint moments was accomplished using Ortho Track 6.6.4 software. Hip and knee joint ROM were calculated by subtracting the minimum joint angle during the gait cycle from the maximum joint angle achieved during the swing phase. Ankle joint ROM was calculated by subtracting the minimum ankle joint angle at the toe off point from the maximum ankle joint angle during the gait cycle. The vertical ground reaction force data were scaled by body weight. The values for the first peak, developed at the instant of loading response, and the second peak, developed at the time of push-off (onset of forward propulsion), were averaged across trials for each subject.

We applied sEMG muscle effort, defined as the percentage of maximum voluntary contraction (%MVC) averaged across all participants. %MVC was calculated using the following equation: % MVC = [(EMGtask − EMGresting)/(EMGmax − EMGresting)] × 100 (%)^74^. The MVC test is the normalizing method proposed by the SENIAM and Kinesiology guidelines and is the most widely used normalization method^88^. To normalize the sEMG signal amplitude, three 5-s maximal muscle contraction measurement trials were performed with each muscle to determine each participant’s MVC. To reduce movement artifacts, a sampling frequency of 1000 Hz was used, and raw sEMG signals were bandpass filtered between 10 and 350 Hz and full-wave-rectified with Noraxon software (MyoResearch XP Master Edition). In addition, the root mean squared values of the signals were calculated using a sliding 100-ms window for analysis. The data were passed through a sixth-order Butterworth low-pass filter with a 6-Hz cutoff frequency to create a linear envelope and normalized to the MVC data obtained prior to the tasks. The average normalized sEMG activity was processed within the selected phases of the gait cycle using MATLAB software (MathWorks, Inc., Natick, MA, USA)^89^. Individual gait cycles were determined using the footswitch data, where each stride was considered the period between successive heel-strikes by the same leg. The sEMG patterns for each stride were time normalized and expressed as a percentage of the total gait cycle (0–100%).

Baseline cardiopulmonary metabolic energy efficiency was measured in a comfortable standing position for 5 min. Then, the metabolic energy cost was recorded during 6 min of treadmill walking at each participant’s most comfortable gait speed. Participants identified their most comfortable gait speeds by walking at their own pace for 3 min without using handrails. The metabolic energy cost test at all time points was performed on the same treadmill at the same speed and using the same measurement method. The net cardiopulmonary metabolic cost (mL/kg/min) was calculated by subtracting the standing oxygen demand (baseline) from the average oxygen uptake during the last 2 min.

### Statistics

Statistical analyses were performed with SPSS version 22.0 (IBM, Armonk, NY, USA), and the significance level was set at 0.05. Descriptive statistics are expressed as mean (standard deviation, SD). To determine the appropriate statistical tests, we checked the normal distribution of the data, based on which we applied parametric tests. We used independent t tests for continuous variables and χ^2^ tests for categorical variables to compare participants' baseline characteristics between the experimental and control groups. Repeated measures ANOVA was used to examine the main effects of exercise over time in both groups (experimental and control group) and time points (Mid, Post, 1 m FU, and 3 m FU). Post-hoc tests determined whether changes from baseline were significant at Mid, Post, 1 m FU, and 3 m FU in each group, and the significance levels of those tests were adjusted using Bonferroni correction.

## Supplementary Information

Supplementary Information.

## Data Availability

The datasets generated during and/or analyzed during the current study are available from the corresponding author on reasonable request.
