# Supplementary material for: Exercise with a wearable hip-assist robot improved physical function and walking efficiency in older adults
Source: Sci Rep. 2023 May 4;13:7269. doi: 10.1038/s41598-023-32335-8 (PMC10160081; doi:10.1038/s41598-023-32335-8)
Supplement: Supplementary file 1 — Supplementary Information. [file 41598_2023_32335_MOESM1_ESM.docx]

**Supplementary Information**

**Exercise with a Wearable Hip-assist Robot Improved Physical Function and Walking Efficiency in Older Adults**

Su-Hyun Lee, Jihye Kim, Bokman Lim, Hwang-Jae Lee, Yun-Hee Kim

**Corresponding author**

Yun-Hee Kim, MD, PhD

Department of Physical and Rehabilitation Medicine, Center for Prevention and Rehabilitation, Heart Vascular Stroke Institute, Samsung Medical Center, Sungkyunkwan University School of Medicine, Seoul, 06351, Republic of Korea

Department of Health Science and Technology, Department of Medical Device Management and Research, SAIHST, Sungkyunkwan University, Seoul, 06355, Republic of Korea

Email: yun1225.kim@samsung.com, yunkim@skku.edu

**Co-corresponding author**

Hwang-Jae Lee, PhD

Department of Physical and Rehabilitation Medicine, Center for Prevention and Rehabilitation, Heart Vascular Stroke Institute, Samsung Medical Center, Sungkyunkwan University School of Medicine, Seoul, 06351, Republic of Korea

Robot Business Team, Samsung Electronics, Suwon, 16677, Republic of Korea

Email: hwangjae.lee@samsung.com

**EX1 assistance algorithm**

**Delayed output feedback control (DOFC) for gait assistance**

As shown in Supplementary Fig. S1(A), this time-delayed, self-excited feedback control method does not include a gait phase estimator or references for generating assistive torques. The assistive torques are immediately applied following the movement of the user by updating the change of hip motion at every control period (100Hz). Supplementary Fig. S1(B) shows the control flow of the DOFC-based assistance.

**1. Smoothing hip sensor data with a low-pass filter (LPF)**

The left and right hip angles *q_r_*(*t*), *ql*(*t*) are obtained from the angular sensors located at the hip joints. We then calculate the state variable *y_raw_* = sin *q_r_*(*t*) − sin *q_l_*(*t*) to represent the current gait behavior. The original control state *y_raw_* is smoothed by passing through a first-order low-pass filter:

*y^cur^* = (1 − *α*)*y^prv^* + *αy^cur^ ,* (0 *< α <* 1) (1)

*raw*

where *y^cur^* is the currently smoothed state value, *y^prv^* is the previously smoothed hip angle, *y^cur^* is the currently sensed original state, and *α* is the smoothing factor. The smoothing factor *α* = 0*.*05 (cut-off frequency *f_c_* ≈ 0*.*84 Hz) in Eq. (1) was selected to generate the smoothed output torque.

*raw*

**2. Assistive torque from delayed state feedback**

Using the defined output state *y*(*t*), the assistance torque *τ* is generated from a combination of appropriate time delays ∆*t* and control gain *κ.*

*τ* (*t*) = *κy*(*t* − ∆*t*) (2)

where ∆*t* is the time delay, and *κ* is the feedback gain.

**3. Synergetic hip extension and flexion assistance**

The basic assistance strategy (Supplementary Fig. S1(B)) can be extended for both right/left hip assistive torque generation *τ_r,des_*, *τ_l,des._* as the following form:

Right hip flexion, left hip extension assistance:

*τ_r,des_*(*t*) = −*τ* (*t*) (3)

*τ_l,des_*(*t*) = *τ* (*t*)

Left hip flexion, right hip extension assistance:

*τ_l,des_*(*t*) = *τ* (*t*) (4)

*τ_r,des_*(*t*) = −*τ* (*t*)

The same hip extension and flexion torques are simultaneously generated for (propulsive) gait synergy.

**4. Tunable control parameters of DOFC-based assistance**

In this controller, the two main control parameters are time-delay ∆*t* and feedback gain *κ.* By increasing or decreasing the default time-delay value ∆*t* = 0*.*25 s in Eq. (2), we can smoothly adjust the assistance response. For example, with the maximum swing speed as a reference point (near the intersection of both legs), maximum assistance may be generated beforehand (early timing) or later (late timing). The self-selected walking speed of the elderly was slow, and the default value of 0.25 was used in most cases. If the walking speed is over 4 km/h, it may be necessary to reduce it to 0.2 s for fast response. We can also adjust the strength (strong or weak) by adjusting the default feedback gain *κ* = 7*.*0. As shown in Supplementary Fig. S2, we can fine-tune the assist intensity linearly for each patient condition by adjusting the gain value (See the relationship between the root means square (RMS) torque value and gain in Fig. S2(B).

**5. Seamless assistance in changing walking speed**

Supplementary Figure S3(A) illustrates how the assistance torque profile changed as the walking speed changed with fixed delay and gain parameters (∆*t* = 0.25 s, *κ* = 8). Human walking requires higher hip power (or torque) as walking speed increases. Therefore, generating a larger assistive power when required may be a desirable characteristic. The large positive (assistive) power compared to the small negative (resistive) power in Supplementary Fig. S3(B) indicates that the DOFC controller can operate under various speed conditions with minimum human-exoskeleton mismatch.

**6. Torque changes according to various environmental changes**

The time-delay feedback control-based algorithm can flexibly respond to different physical activities. This is because it does not require a separate task recognizer and does not depend on recognizer accuracy. Our method reflects changes in hip pattern when the walking speed or environment changes in real-time (100 Hz) relative to the input torque. Supplementary Fig. S4 shows how the torque magnitude and shape change during walking uphill/downhill, level ground, and up/downstairs. Supplementary Fig. S5 shows the relationships between the left and right hip angle changes and the assistive torque profile when walking speed changes. This assistive control strategy achieves stable interactive control by naturally and seamlessly increasing torque when climbing stairs or walking at a high speed and reducing torque when walking downhill or at a low speed.

**7. Torque trajectory generated by the device**

The default time-delay and feedback gain values are 7 and 0.25 sec, respectively. During training, the physical therapist adjusted the settings by reflecting the user's opinion. With a time-delay, the default value of 0.25 s was used in most cases, while 0.20 s was used only for subjects with a fast walking speed (about 5.0 km/h or more). Feedback gain was based on user comfort and was set prior to the onset of discomfort. Feedback gain was set at values of 6 to 8, where the maximum assistance torque generated was about 4 to 5.5 Nm. Supplementary Fig. S6 shows the torque trajectory generated by the device during self-selected walking training in the default settings of time-delay of 0.25 s and gain of 7."


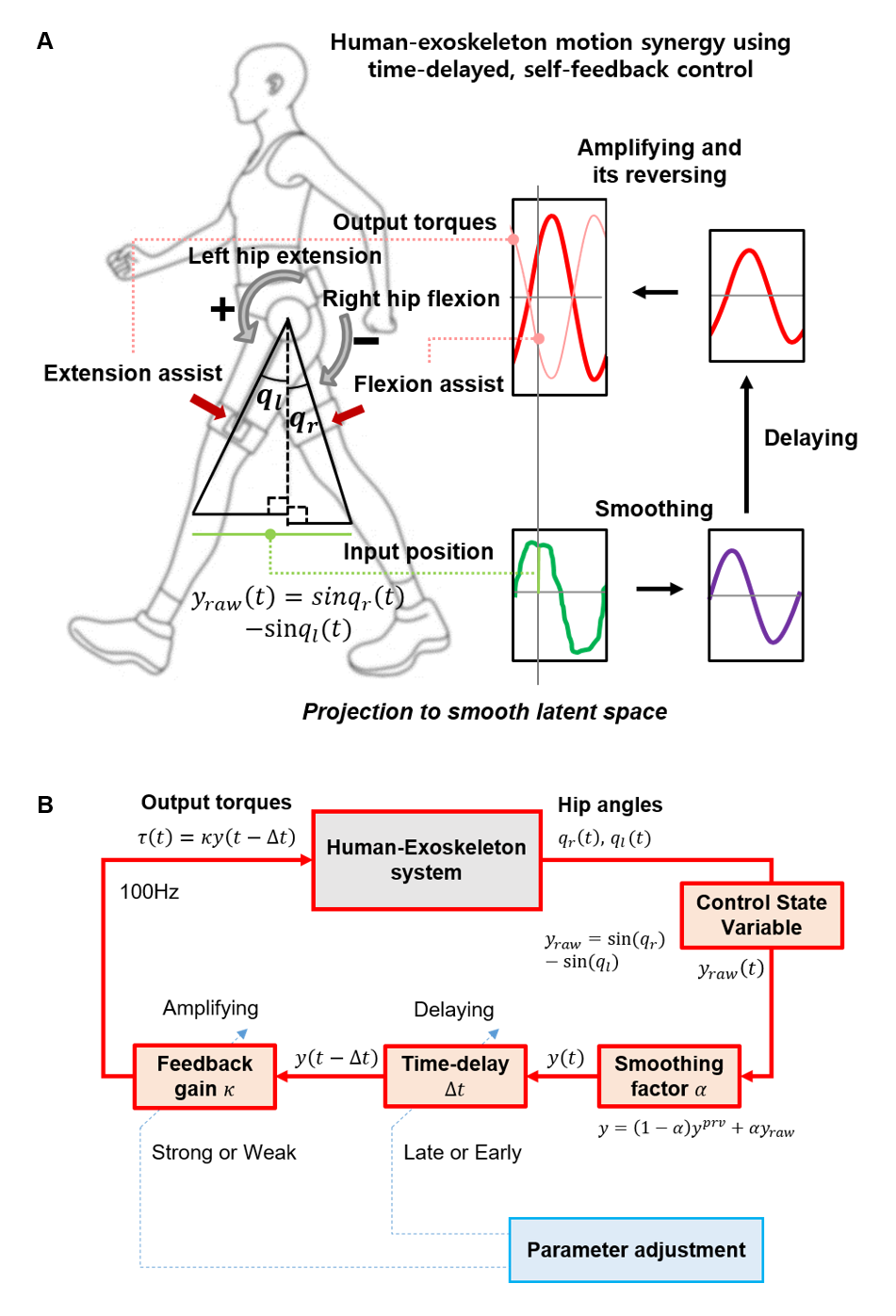


**Supplementary Figure S1. Gait assistance strategy with delayed output feedback control.** *q_r_* and ql are the right and left hip angles, respectively. ∆t is the time-delay and κ is the feedback gain. **(A)** Hip assistance strategy. **(B)** Self-excited feedback control loop.


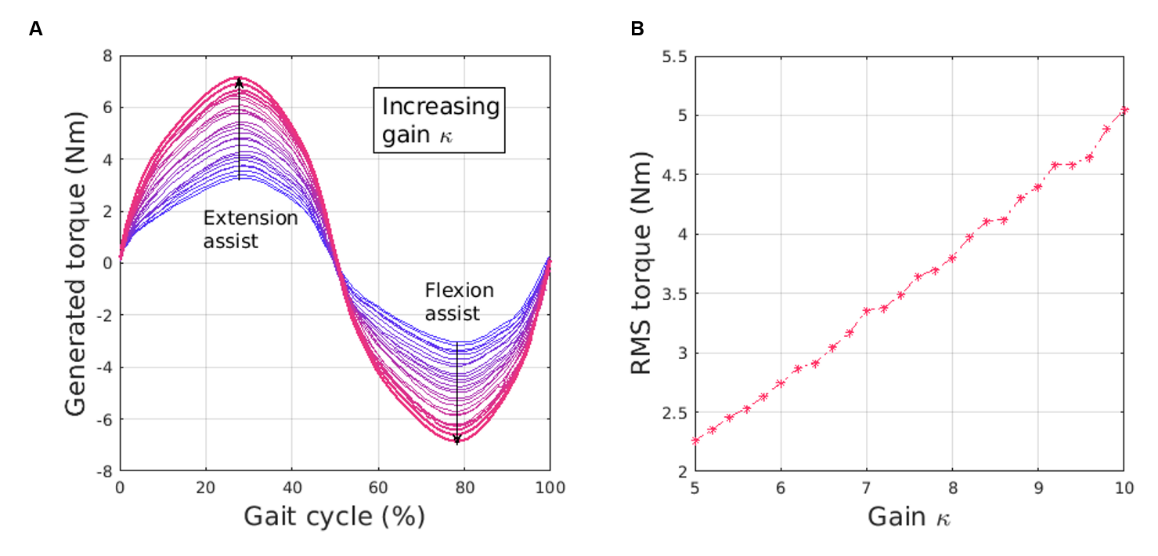


**Supplementary Figure S2. Torque changes generated by input gain changes.** The feedback gain κ was increased from 5 to 10 with 0.2 increments for every two steps (1 walking cycle). The treadmill walking speed is 4 km/h and the time-delay ∆t is fixed to 0.25 s. **(A)** Generated torque. **(B)** RMS torque.


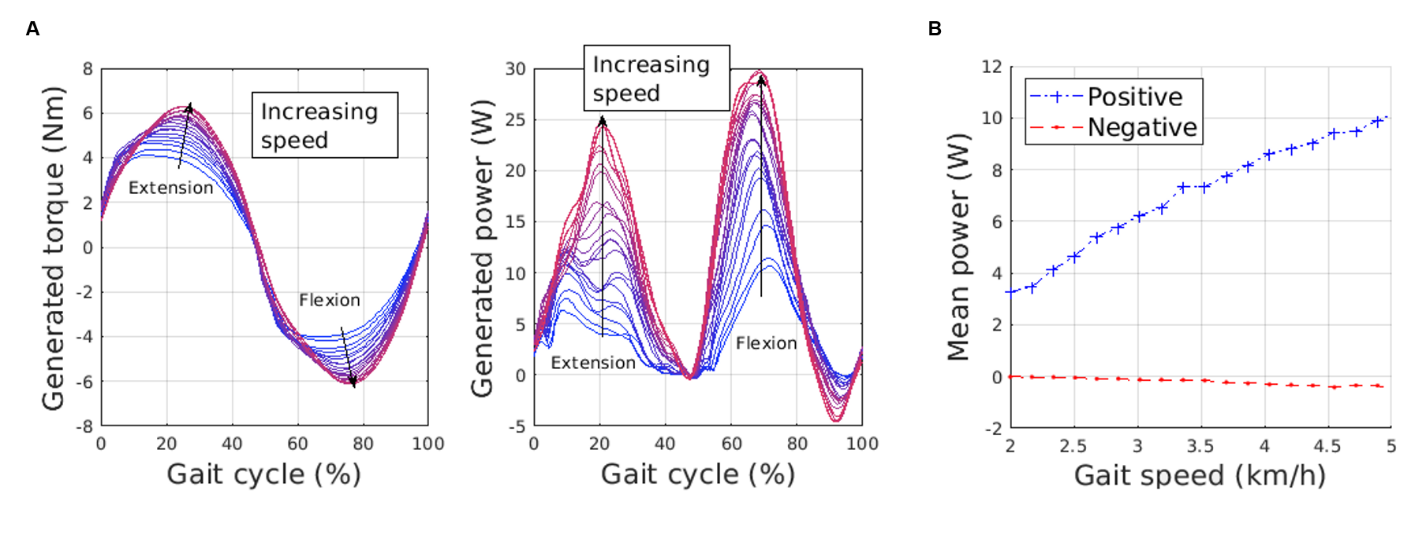


**Supplementary Figure S3. Torque and power changes generated by walking speed changes.** The treadmill speed was increased from 2 to 5 km/h with fixed control parameters ∆t = 0.25 s, κ = 8. Positive: mean positive power, Negative: mean negative power. **(A)** Generated torque and power. **(B)** Mean power.

*
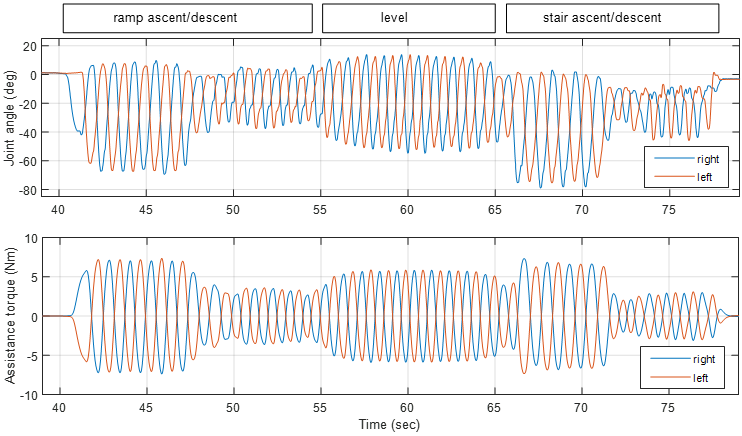
*

**Supplementary Figure S4. Torque changes according to various environmental changes, with a fixed time-delay of 0.25 s and feedback gain of 8.0.**

*
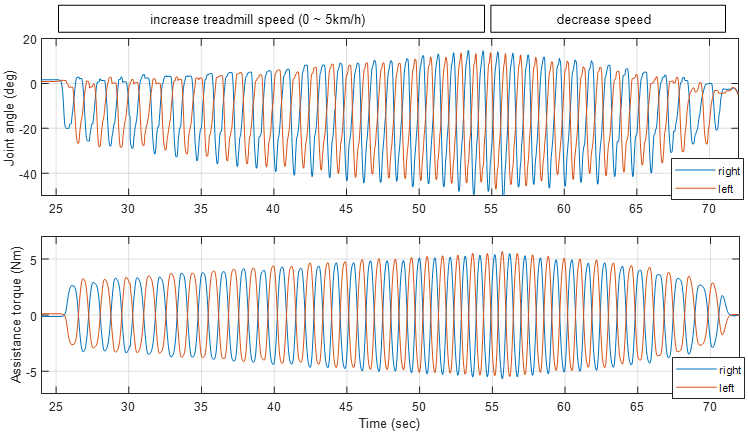
*

**Supplementary Figure S5. Torque changes caused by speed changes with a fixed time-delay of 0.25 s and a feedback gain of 8.0.** The stopped treadmill is increased to a maximum speed of 5 km/h and decelerated back to stopped.

**

**Supplementary Figure S6. Examples of hip angle, angular velocity, and torque trajectory collected by the device during actual gait training.** The default time-delay and feedback gain were 0.25 s and 7, respectively.

*
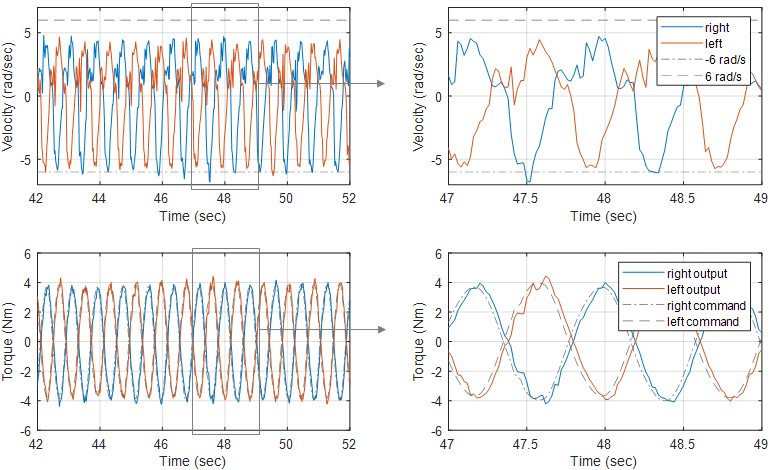
*

**Supplementary Figure S7. Torque tracking performance at fast hip angular velocity.** The root mean squared error (RMSE) between the command torque and the output torque at 5 km/h treadmill walking is about 0.6 Nm (time delay 0.15, feedback gain 7).


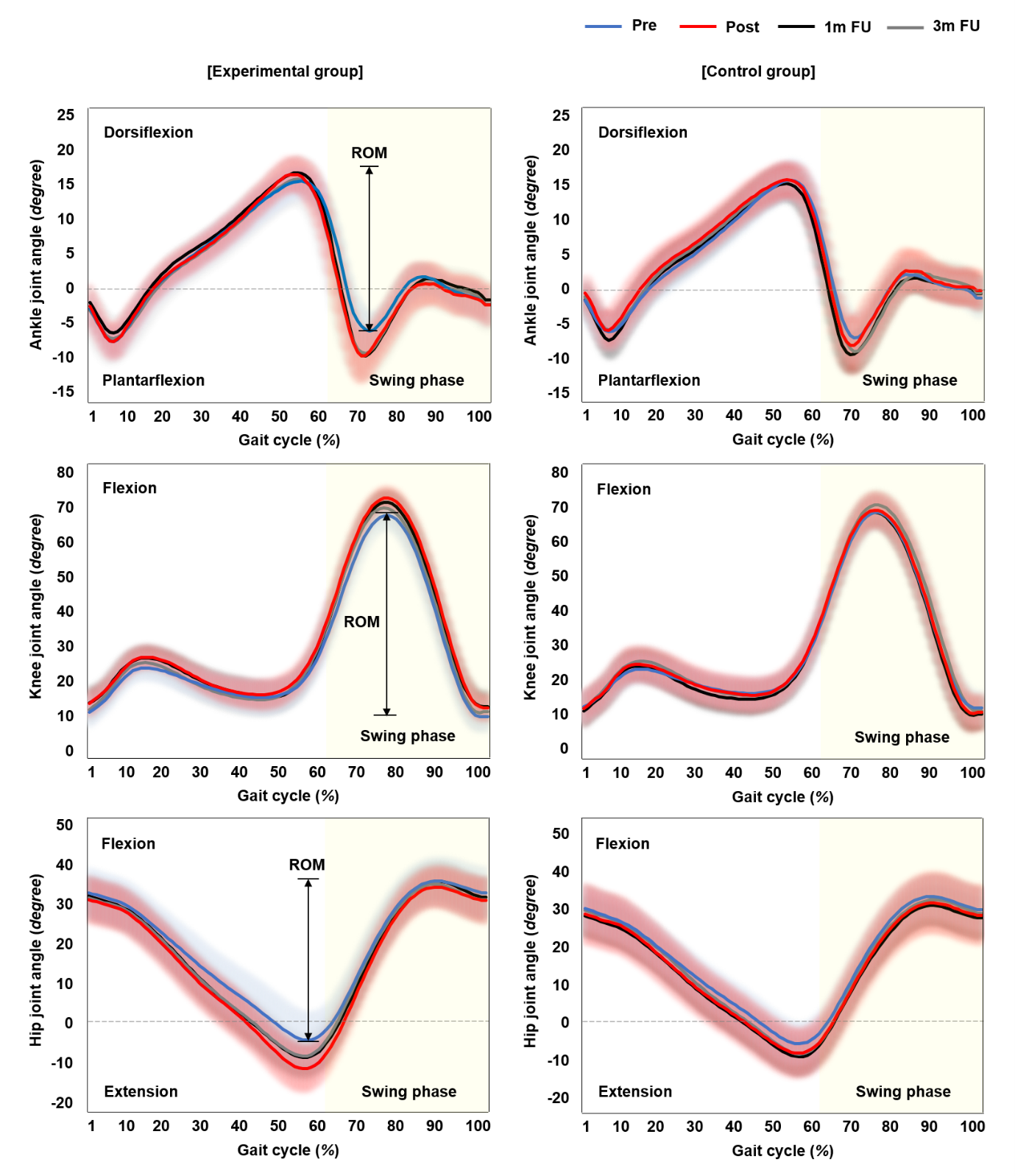


**Supplementary Figure S8. Joint angle.** Diagrams showing differences in mean joint angle during the gait cycle (%) at Pre, Post, 1m, and 3m FU in the experimental and control groups.

FU: follow up, ROM: range of motion


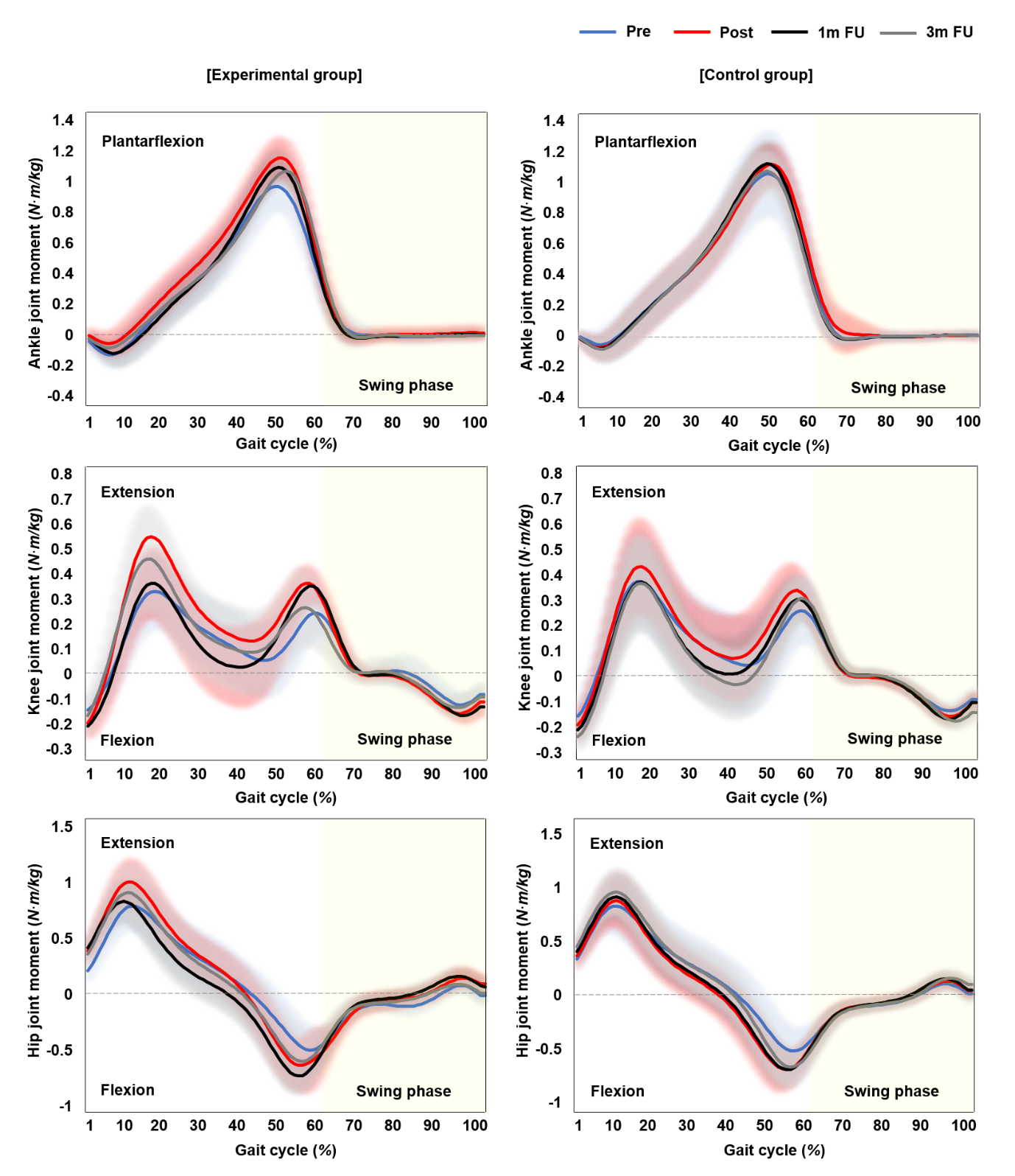


**Supplementary Figure S9. Joint moment.** Diagrams showing differences in mean joint moment during the gait cycle (%) at Pre, Post, 1m, and 3m FU in the experimental and control groups.

FU: follow up


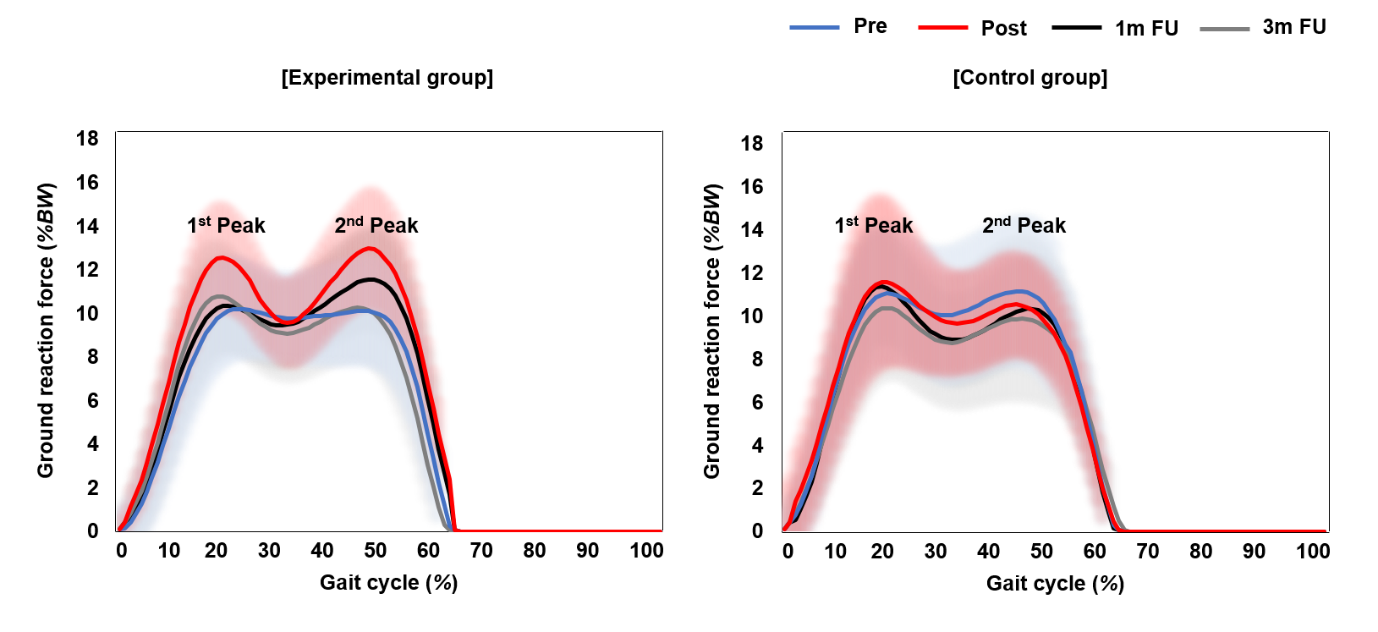


**Supplementary Figure S10. Vertical ground reaction force.** Diagrams showing differences in mean ground reaction force during the gait cycle (%) at Pre, Post, 1m, and 3m FU in the experimental and control groups.

FU: follow up


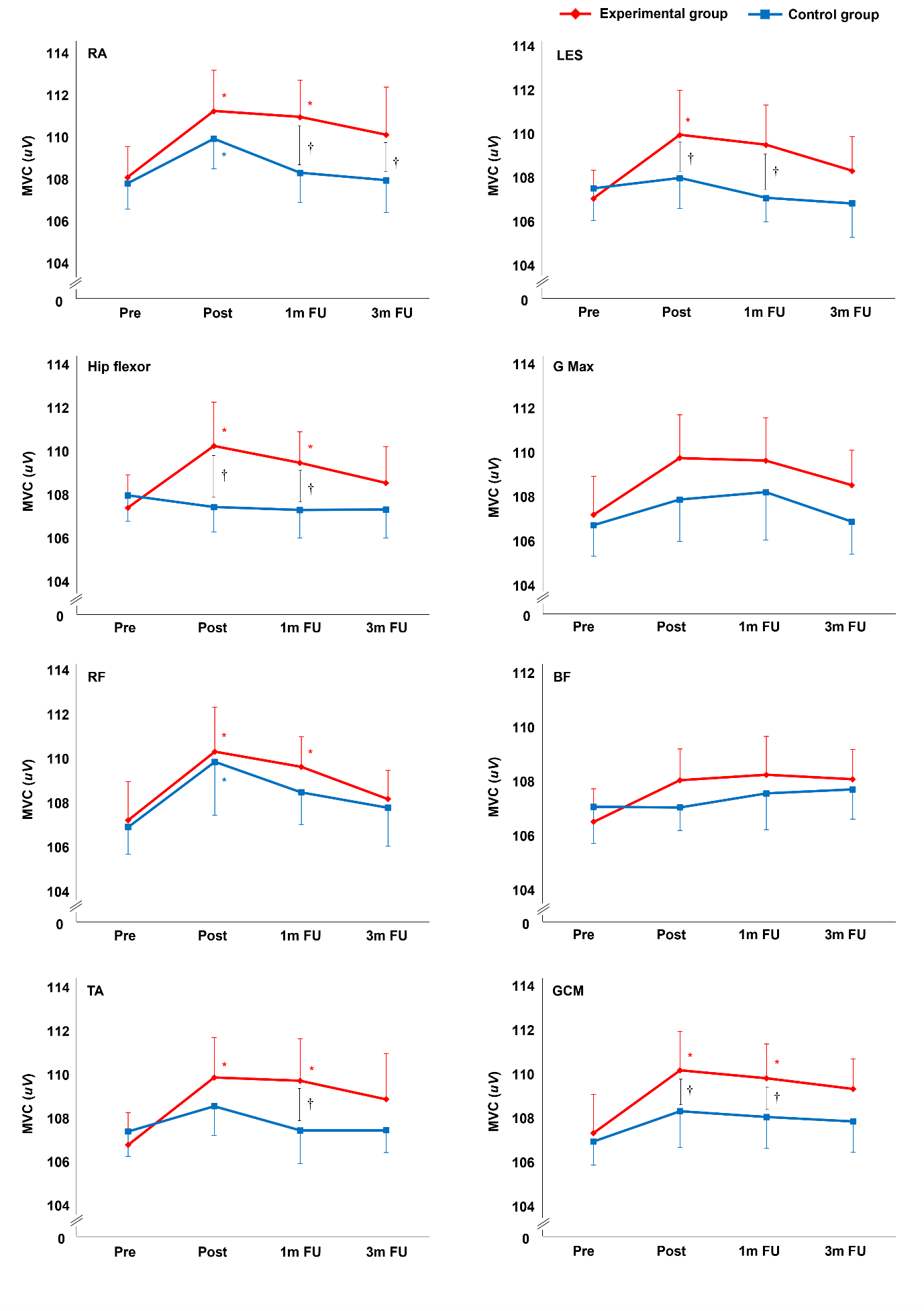


**Supplementary Figure S11. Maximum voluntary contraction.** Changes in MVC at Pre, Post, 1m, and 3m FU in the experimental and control groups and effects of clinical measures between the experimental and control groups.

FU: follow up, MVC: maximum voluntary contraction, RA: rectus abdominis, LES: lumbar extensor spinae, G Max: gluteus maximus, RF: rectus femoris, BF: biceps femoris, TA: tibialis anterior, GCM: gastrocnemius medialis

^*^Significant change compared with Pre (P < 0.05), ^†^Independent t test (P < 0.05)

**Supplementary Table S1. Effect of EX1 exercise on muscle effort (%MVC)**

|  | Experimental group | | | |  | Control group | | | | Between groups |
| --- | --- | --- | --- | --- | --- | --- | --- | --- | --- | --- |
|  | Pre | Post | 1m FU | 3m FU |  | Pre | Post | 1m FU | 3m FU | *P* value |
| RA | 7.18(1.34) | 6.88(0.62)^*^ | 7.01(0.74) | 7.05(1.38) |  | 7.70(1.47) | 7.84(2.82) | 7.73(2.13) | 7.73(1.53) | 0.001 |
| LES | 26.09(4.97) | 25.09(4.87)^**^ | 25.22(4.29)^**^ | 25.41(3.57)^*^ |  | 25.15(4.68) | 24.71(5.30) | 25.03(5.82) | 24.90(4.78) | 0.448 |
| Hip flexor | 14.46(3.49) | 13.44(2.33)^**^ | 13.65(3.78)^**^ | 14.04(4.67) |  | 13.89(3.74) | 14.02(3.80) | 14.20(4.03) | 14.58(4.23) | 0.580 |
| G Max | 21.73(5.47) | 21.30(5.97) | 21.29(6.66) | 21.56(5.65) |  | 22.80(6.83) | 21.71(7.90) | 22.44(7.67) | 22.33(6.01) | 0.339 |
| RF | 19.91(5.87) | 18.52(7.97)^*^ | 18.73(7.64) | 18.83(6.66) |  | 19.87(6.26) | 18.89(8.39)^*^ | 19.21(7.53) | 19.23(6.35) | 0.751 |
| BF | 17.66(5.98) | 16.76(8.22)^*^ | 16.77(9.96) | 17.25(6.46) |  | 18.18(7.13) | 17.41(9.26) | 17.47(9.86) | 17.64(8.24) | 0.620 |
| TA | 20.78(7.52) | 19.08(6.90)^**^ | 19.37(5.22)^*^ | 19.93(3.91) |  | 20.70(6.58) | 19.85(6.57)^**^ | 19.86(7.84)^*^ | 20.36(9.01) | 0.667 |
| GCM | 22.78(12.58) | 21.86(10.04)^*^ | 21.91(12.21)^*^ | 22.20(13.19)^*^ |  | 23.03(10.35) | 22.74(9.25) | 22.71(9.12) | 22.77(10.53) | 0.687 |

Data are expressed as mean (standard deviation).

^*^Significant change compared with Pre (P < 0.05), ^**^Significant change compared with Pre (P < 0.01)

FU: follow up, MVC: maximum voluntary contraction, RA: rectus abdominis, LES: lumbar extensor spinae, G Max: gluteus maximus, RF: rectus femoris, BF: biceps femoris, TA: tibialis anterior, GCM: gastrocnemius medialis

**Supplementary Table S2. Effect of EX1 exercise on maximum voluntary contraction**

|  | Experimental group | | | |  | Control group | | | | Between groups |
| --- | --- | --- | --- | --- | --- | --- | --- | --- | --- | --- |
|  | Pre | Post | 1m FU | 3m FU |  | Pre | Post | 1m FU | 3m FU | *P* value |
| RA | 108.13(2.91) | 111.28(3.88)^*^ | 110.99(3.51)^*^ | 110.15(4.52) |  | 107.84(2.46) | 109.96(2.86)^*^ | 108.34(2.83) | 107.98(3.06) | 0.001 |
| LES | 107.09(2.54) | 109.99(4.05)^*^ | 109.53(3.65) | 108.34(3.11) |  | 107.55(2.94) | 108.02(2.78) | 107.11(2.19) | 106.85(3.09) | 0.000 |
| Hip flexor | 107.43(3.04) | 110.27(4.06)^*^ | 109.50(2.87)^*^ | 108.57(3.35) |  | 107.99(2.38) | 107.46(2.30) | 107.32(2.60) | 107.35(2.62) | 0.000 |
| G Max | 107.22(3.47) | 109.78(3.91) | 109.66(3.87) | 108.55(3.17) |  | 106.75(2.80) | 107.90(3.79) | 108.24(4.34) | 106.91(2.94) | 0.005 |
| RF | 107.25(3.49) | 110.34(4.02)^*^ | 109.66(2.70)^*^ | 108.21(2.58) |  | 106.94(2.46) | 109.88(4.79)^*^ | 108.51(2.90) | 107.81(3.45) | 0.235 |
| BF | 106.54(2.44) | 108.07(2.31) | 108.27(2.83) | 108.11(2.20) |  | 107.09(2.72) | 107.07(1.70) | 107.59(2.68) | 107.73(2.18) | 0.230 |
| TA | 106.81(2.96) | 109.90(3.65)^*^ | 109.75(3.84)^*^ | 108.90(4.20) |  | 107.42(2.28) | 108.58(2.68) | 107.48(3.05) | 107.48(2.04) | 0.016 |
| GCM | 107.35(3.52) | 110.20(3.54)^*^ | 109.84(3.12)^*^ | 109.36(2.75) |  | 106.97(2.15) | 108.35(3.30) | 108.08(2.84) | 107.88(2.79) | 0.002 |

Data are expressed as mean (standard deviation).

^*^Significant change compared with Pre (P < 0.05)

FU: follow up, MVC: maximum voluntary contraction, RA: rectus abdominis, LES: lumbar extensor spinae, G Max: gluteus maximus, RF: rectus femoris, BF: biceps femoris, TA: tibialis anterior, GCM: gastrocnemius medialis

**Supplementary Table S3. Effect of EX1 exercise on functional assessment**

|  | Experimental group | | | | |  | Control group | | | | | Between groups |
| --- | --- | --- | --- | --- | --- | --- | --- | --- | --- | --- | --- | --- |
|  | Pre | Mid | Post | 1m FU | 3m FU |  | Pre | Mid | Post | 1m FU | 3m FU | *P* value |
| 10MWT-SSV (m/s) | 1.21(0.16) | 1.32(0.17)^*^ | 1.36(0.18)^*^ | 1.35(0.16)^*^ | 1.34(0.17)^*^ |  | 1.18(0.18) | 1.22(0.18) | 1.26(0.14)^*^ | 1.22(0.15) | 1.21(0.19) | 0.009 |
| 10MWT-FV (m/s) | 1.48(0.25) | 1.61(0.23)^*^ | 1.63(0.24)^*^ | 1.61(0.19)^*^ | 1.62(0.23)^*^ |  | 1.42(0.23) | 1.46(0.25) | 1.49(0.22)^*^ | 1.44(0.20) | 1.43(0.23) | 0.012 |
| BBS | 51.87(1.78) | 53.37(2.06)^**^ | 54.73(1.14)^**,§§^ | 54.63(1.47)^**,§^ | 54.47(1.76)^**,§^ |  | 52.53(1.33) | 53.70(1.44)^**^ | 54.30(1.18)^**^ | 54.30(1.26)^**^ | 54.03(1.25)^**^ | 0.902 |
| FSST (sec) | 9.69(1.63) | 8.01(1.74)^**^ | 7.48(1.42)^**,§^ | 7.48(0.83)^**^ | 7.46(0.89)^**^ |  | 9.64(1.76) | 9.22(2.07)^*^ | 8.72(1.80)^*^ | 8.92(2.38)^*^ | 9.04(2.68) | 0.012 |
| TUG (sec) | 9.75(1.85) | 8.89(1.26)^*^ | 8.58(1.18)^*^ | 8.68(1.09)^*^ | 8.74(1.23)^*^ |  | 10.09(1.93) | 9.53(1.52) | 9.30(1.25)^*^ | 9.67(1.56) | 9.77(1.70) | 0.032 |
| FRT (cm) | 24.73(6.36) | 28.09(5.96)^*^ | 29.47(6.08)^**^ | 28.87(4.54)^**^ | 28.70(4.29)^**^ |  | 24.86(4.92) | 26.48(5.06)^*^ | 27.43(4.54)^*^ | 27.35(5.59)^*^ | 26.97(5.27)^*^ | 0.255 |
| SPPB | 8.80(0.55) | 10.80(1.19)^**^ | 11.43(0.90)^**,§^ | 11.53(0.86)^**,§^ | 11.60(0.72)^**,§^ |  | 8.87(0.57) | 10.83(0.87)^**^ | 11.30(0.75)^**,§^ | 11.47(0.78)^**,§^ | 11.53(0.90)^**,§^ | 0.843 |
| GDS | 1.97(2.71) | 0.97(1.79)^*^ | 0.90(1.65)^*^ | 1.07(1.66) | 1.30(2.02) |  | 1.37(2.16) | 0.97(1.75) | 1.00(2.30) | 1.07(1.53) | 1.40(2.27) | 0.862 |

Data are expressed as mean (standard deviation).

^*^ *Significant change compared with Pre (P < 0.05), **Significant change compared with Pre (P < 0.01)

§Significant change compared with Mid (P < 0.05), §§Significant change compared with Mid (P < 0.01)

FU: follow up, 10MWT-SSV: 10-Meter Walk Test for self-selected walking velocity, 10MWT-FV: 10-Meter Walk Test for fastest walking velocity, BBS: Berg Balance Scale, FSST: Four Square Step Test, TUG: Timed Up and Go, FRT: Functional Reach Test, SPPB: Short Physical Performance Battery, GDS: Geriatric Depression Scale
